# The *Pseudomonas putida* CsrA/RsmA homologues negatively affect c‐di‐GMP pools and biofilm formation through the GGDEF/EAL response regulator CfcR

**DOI:** 10.1111/1462-2920.13848

**Published:** 2017-07-21

**Authors:** Óscar Huertas‐Rosales, Manuel Romero, Stephan Heeb, Manuel Espinosa‐Urgel, Miguel Cámara, María Isabel Ramos‐González

**Affiliations:** ^1^ Department of Environmental Protection Estación Experimental del Zaidín, CSIC Profesor Albareda, 1, Granada 18008 Spain; ^2^ Centre for Biomolecular Sciences, School of Life Sciences University of Nottingham Nottingham NG7 2RD UK

## Abstract

Expression of *cfcR*, encoding the only GGDEF/EAL response regulator in *Pseudomonas putida*, is transcriptionally regulated by RpoS, ANR and FleQ, and the functionality of CfcR as a diguanylate cyclase requires the multisensor CHASE3/GAF hybrid histidine kinase named CfcA. Here an additional level of *cfcR* control, operating post‐transcriptionally via the RNA‐binding proteins RsmA, RsmE and RsmI, is unraveled. Specific binding of the three proteins to an Rsm‐binding motif (5′CANGGANG3′) encompassing the translational start codon of *cfcR* was confirmed. Although RsmA exhibited the highest binding affinity to the *cfcR* transcript, single deletions of *rsmA*, *rsmE* or *rsmI* caused minor derepression in CfcR translation compared to a *ΔrsmIEA* triple mutant. RsmA also showed a negative impact on c‐di‐GMP levels in a double mutant *ΔrsmIE* through the control of *cfcR*, which is responsible for most of the free c‐di‐GMP during stationary phase in static conditions. In addition, a CfcR‐dependent c‐di‐GMP boost was observed during this stage in *ΔrsmIEA* confirming the negative effect of Rsm proteins on CfcR translation and explaining the increased biofilm formation in this mutant compared to the wild type. Overall, these results suggest that CfcR is a key player in biofilm formation regulation by the Rsm proteins in *P. putida*.

## Introduction

Proteins belonging to the CsrA/RsmA (acronyms for carbon storage regulator and regulator of secondary metabolism) family are small sequence‐specific RNA‐binding regulators that activate or repress gene expression by altering translation, RNA stability and/or transcript elongation (Romeo *et al*., 2013). They are present in diverse Gram‐negative and Gram‐positive bacteria (Ulrich and Zhulin, 2010). CsrA was first described in *Escherichia coli* (Romeo *et al*., 1993; Romeo, 1998), where it plays a major role in controlling the intracellular carbon flux, by negatively regulating glycogen metabolism and several enzymes involved in central carbohydrate metabolism (Sabnis *et al*., 1995; Yang *et al*., 1996). In *Pseudomonas protegens* CHA0 (previously *Pseudomonas fluorescens*) the CsrA homologues, RsmA and RsmE, not only control metabolism but also the production of biocontrol‐related traits (Reimmann *et al*., 2005). Furthermore, CsrA/RsmA systems have been shown to play a key role in the control of virulence through complex regulatory networks (Vakulskas *et al*., 2015).

The activity of RsmA/CsrA has been shown to be modulated by small regulatory RNA molecules that can bind these regulators with high affinity titrating them out and preventing them from binding to their target mRNAs. These include the sRNAs RsmY, RsmW and RsmZ in *P. aeruginosa* (Kay *et al*., 2006; Miller *et al*., 2016) and RsmX, RsmY and RmsZ in *P. protegens* (Heeb *et al*., 2002; Kay *et al*., 2005). The RsmA family proteins and their cognate small RNAs are part of the GacS/GacA signal transduction pathway. It is known that titration of RsmA by the sRNAs increases bacterial attachment and biofilm formation, whereas excess of Rsm proteins promote the planktonic lifestyle of these bacteria; the later functions in opposition to that of the second messenger c‐di‐GMP, an increase in which leads to cellular aggregation (Römling *et al*., 2013). Some of the molecular elements connecting the Rsm and c‐di‐GMP regulatory networks are being characterized (Colley *et al*., 2016; Valentini and Filloux, 2016 and references therein).

GGDEF and EAL protein domains are responsible for the synthesis and hydrolysis of c‐di‐GMP through their role in diguanylate cyclase (DGC) and phosphodiesterase (PDE) activities respectively. These activities are key in controlling the turn‐over of this second messenger in bacterial cells (Hengge, 2009; Römling and Simm, 2009). The gene *rup4959*, which encodes the unique response regulator containing both GGDEF and EAL domains in *P. putida* KT2440, was identified as being preferentially expressed in the corn rhizosphere (Matilla *et al*., 2007). When overexpressed, *rup4959* increases the levels of free c‐di‐GMP in the bacterial cells and confers a pleiotropic phenotype that includes enhanced biofilm and pellicle formation capacity, cell aggregation and crinkle colony morphology. In order to trigger the DGC activity, the protein encoded by *rup4959* requires to be phosphorylated at the Asp65 in its REC domain (Matilla *et al*., 2011) and also the multi‐sensor (CHASE3/GAF) hybrid histidine kinase CfcA. Therefore, we have recently renamed Rup4959 to CfcR (Ramos‐González *et al*., 2016).

**Figure 1:**
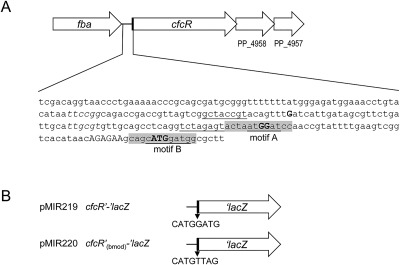
Physical map and constructs involving *cfcR*. A. Features in the promoter, leader and coding sequences of *cfcR*. Transcription initiation points, previously determined experimentally, distal and proximal (double) are indicated in bold as capital ‘G’ (Matilla *et al*., 2011). Two sequences matching the SELEX‐derived consensus for the global posttranscriptional regulator CsrA (RUACARGGAUGU) (Dubey *et al*., 2005) are in grey boxes (motif A, overlapping the proximal transcription initiation points; and motif B, overlapping the start codon). Nucleotides in these boxes coinciding with the consensus are underlined. Putative −35 RpoS‐binding sequences are in italic. Predicted sequences for an extended −10 are underlined. Shine Dalgarno (AGAGAA) and start codon (ATG in bold) are indicated. Diagram not to scale. B. Translational fusions involving the *cfcR* gene. Plasmids pMIR219 and pMIR220 each contain a translational fusion to *‘lacZ* in the vector pMP220‐BamHI (Table 2). The predicted Rsm binding site CATGGATG (motif B) of pMIR219 was replaced by CATGTTAG in pMIR220.

Previous studies focused in the regulation of *cfcR* have highlighted that its transcription is entirely dependent on RpoS and positively modulated by ANR (Matilla *et al*., 2011) and FleQ (Ramos‐González *et al*., 2016). In addition, a post‐transcriptional regulation of *cfcR* has been suggested. Two motifs which share conservation with the SELEX‐derived consensus for CsrA/RsmA binding 5′RUACARGGAUGU3′ (Dubey *et al*., 2005) were identified in the *cfcR* mRNA (Matilla *et al*., 2011). The first (motif A) overlaps with a distal transcription initiation site of the gene and the second (motif B), showing higher similarity to the consensus, encompasses the translation start codon of *cfcR* (Fig. 1A).

The genome of *P. putida* encodes three CsrA/RsmA homologues named RsmA, RsmE and RsmI (Nelson *et al*., 2002; Winsor *et al*., 2016). Although RsmA and RsmE are more related to CsrA than RsmI (Huertas‐Rosales *et al*., 2016), we have found that RsmI still shares predictive secondary structures with CsrA/RsmA/RsmE. All seven possible mutant strains as a result of the deletion of one, two or three *rsm* genes have been generated in *P. putida* previously to this work. The *rsm* triple mutant showed increased biofilm formation, whereas overexpression of RsmE or RsmI resulted in a reduced bacterial attachment (Huertas‐Rosales *et al*., 2016). This suggests that these Rsm proteins may exert a negative regulation upon diguanylate cyclases in this bacterium and that this effect may be mediated via the control of *cfcR* expression.

In this study, we have analyzed the direct interaction between the three Rsm proteins of *P. putida* and specific motifs in the leader sequence and translation initiation of the *cfcR* mRNA and evaluated the role of these proteins on *cfcR* expression and the free pool of c‐di‐GMP. Our results indicate that the influence on biofilm formation observed for Rsm proteins takes place through direct repression of *cfcR*, which results in reduced levels of c‐di‐GMP in the stationary phase. Therefore, we show that CfcR is a central player in Rsm‐controlled biofilm formation in *P. putida*.

## Results

### The Rsm proteins repress the expression of the response regulator CfcR and its transcriptional regulator RpoS

**Figure 2:**
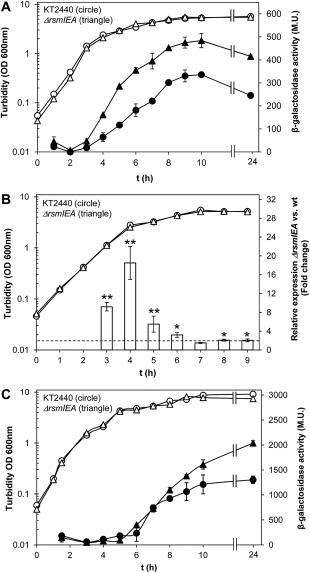
Expression of *cfcR* in the wild type and the triple mutant *ΔrsmIEA*. A. Activity of the translational fusion *cfcR’‐‘lacZ* in pMIR219. Cultures growing in LB supplied with Tc as described in the ‘Experimental procedures’ section were analyzed for turbidity (hollow symbols) and β‐galactosidase activities (solid symbols) at the indicated times. The experiment was performed in triplicate and for each biological replicate activities were assayed in triplicate. Average data and standard deviations are plotted from one representative experiment. Statistically significant differences between wild type and *ΔIEA* β‐galactosidase activities were detected from 4 h onwards (Student's *t* test; *P* < 0.05). B. Time course of the relative quantities of CfcR‐RNA in the triple mutant *ΔIEA* versus the wild‐type strain *P. putida* KT2440. Growth curve of the strains in LB are plotted. Fold changes were based on mRNA measurements obtained with qRT‐PCR. The experiment was carried out in triplicate with three experimental replicates. Average data and standard deviations are plotted. One or two asterisks indicate when results for the triple mutant *ΔIEA* are significantly different from wild type (Student's *t* test, *P* < 0.05 and *P* < 0.01 respectively). A fold change of 2 is indicated with a dotted line. C. Activity of the transcriptional fusion P*_cfcR_::'lacZ* in pMIR200. Samples were analyzed for turbidity (hollow symbols) and β‐galactosidase activities (solid symbols). Experiments and statistical analysis were performed as indicated above for panel A.

To investigate the influence of the Rsm proteins from *P. putida* KT2440 upon the expression of *cfcR*, a translational fusion *cfcR’‐‘lacZ* was generated in pMIR219 (Fig. 1B) and β‐galactosidase activity determined under optimal aeration conditions in the wild‐type strain and a battery of seven mutants hampered in the production of one, two or three Rsm proteins present in this bacterium. These mutants were generated in a previous work (Huertas‐Rosales *et al*., 2016). As expected for an RpoS‐dependent gene (Matilla *et al*., 2011), the expression of *cfcR* is initiated in the transition from the exponential to the stationary phase of growth in the wild type. In the triple *ΔrsmIEA* mutant, *cfcR* expression was activated earlier and enhanced at the onset of stationary phase, an increase that was maintained throughout this phase with levels of β‐galactosidase activity around 1.5 times higher than those of the wild type (Fig. 2A). The deletion of single *rsm* genes caused only minor incremental changes in *cfcR* expression as measured at the advanced stationary phase of growth (Supporting Information Fig. S1A). In the double mutants *ΔrsmIA* and *ΔrsmEA*, a slight increase in expression was also observed at the onset of stationary phase. In the latter strain, this was maintained until further into the stationary phase; as such, the expression pattern of *cfcR* in the double mutant *ΔrsmEA* (Supporting Information Fig. S1B), where only RsmI remains, is most similar to the triple mutant. In the double mutant *ΔrsmIE*, with only RsmA active, a slight decrease in expression was observed at earlier stages of growth. These results suggest a potential gradual relevance of RsmA, RsmE and finally RsmI in the control of *cfcR* expression under the experimental conditions tested.

The relative expression of *cfcR* along the growth curve was also examined by RT‐qPCR in the triple *ΔrsmIEA* mutant versus the wild type, and the results showed higher levels of expression in the mutant. The increase in the relative values (fold change) of mRNA was particularly evident during the exponential phase and reached a peak of ∼18‐fold at the onset of stationary phase, after which it gradually decreased with time (Fig. 2B). Minor effects upon *cfcR* expression were observed in the single *ΔrsmE* and *ΔrsmA* mutants (Supporting Information Fig. S2), whereas transient increases were observed in the double *ΔrmsEA* mutant, where RsmI remained active, and to a lesser extent in the *ΔrsmIA* strain, where only RsmE remained active (Supporting Information Fig. S3). In the double mutant *ΔrsmIE*, with an active RsmA, no differences were observed in the relative mRNA *cfcR* transcripts. Again these results indicated that although the individual loss of Rsm proteins did not have much impact on *cfcR* expression, when RsmA or RsmE remained as unique Rsm proteins, they seem to still exert a major negative effect (RsmA causing more important repression than RsmE). Interestingly, the increase in *cfcR* expression took place at earlier stages of growth as the number of deleted *rsm* genes increased, which is in agreement with the progress in biofilm formation previously described for the triple mutant (Huertas‐Rosales *et al*., 2016).

We hypothesized that the significant enhancement in *cfcR* transcripts observed in the *ΔrsmIEA* strain might perhaps be due to a modified expression pattern of the alternative transcription factor RpoS in this mutant, as a consequence of the release of repression by Rsm proteins upon RpoS. This could also explain that in a transcriptional *cfcR’::'lacZ* fusion, there still remained a positive effect of deleting these post‐transcriptional regulators (Fig. 2C). To investigate this possibility, the expression of RpoS was also analyzed in the seven mutants hampered in one, two and three Rsm proteins, using a translational *rpoS’‐‘lacZ* fusion in the plasmid pMAMV21 (Matilla *et al*., 2011). Expression of *rpoS* differed only in *ΔrsmIEA* background right at the beginning of the stationary phase, when a significant increase was observed in the mutant (Supporting Information Fig. S4). This may explain at least in part the transient accumulation of *cfcR* mRNA observed in the triple mutant *ΔrsmIEA* compared to the wild type. Moreover, *rpoS* has been identified as a target of Rsm proteins in RIP‐seq experiments (our unpublished results) as an indication that RpoS regulation by Rsm proteins is direct. In addition to this negative regulation of RpoS by Rsm proteins, the possibility remained that these proteins directly repress the expression of *cfcR* and/or cause a reduction in its mRNA stability.

### Binding of the Rsm proteins to the *cfcR* transcripts

**Figure 3:**
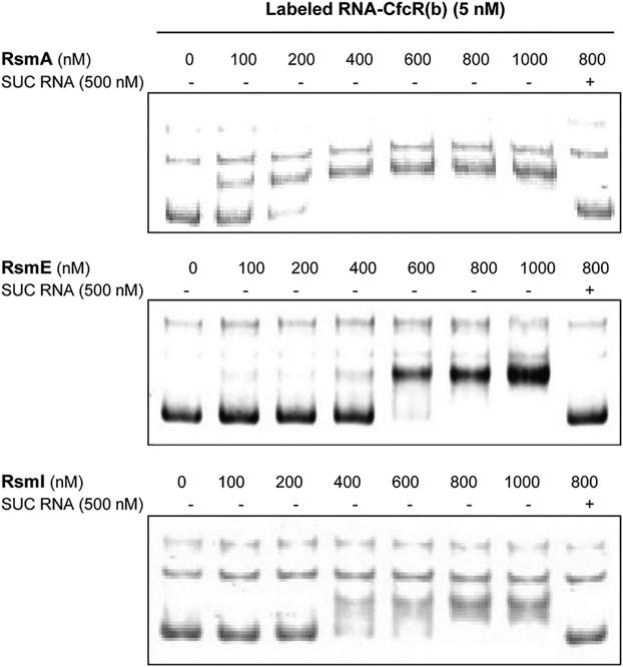
Fluorescence‐based EMSA of RsmA, RsmE and RsmI binding to RNA‐CfcR(b). RNA‐CfcR(b) includes the predicted Rsm binding motif B (see Supporting Information Fig. S5). Note that specific unlabeled competitor (SUC) RNA‐CfcR(b) prevented the formation of the labeled RNA‐CfcR(b)–protein complexes.

**Figure 4:**
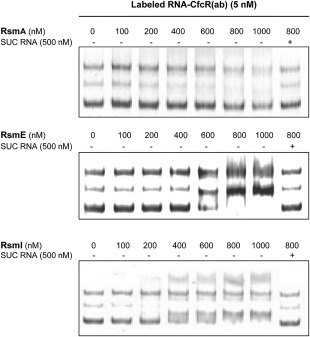
Fluorescence‐based EMSA of RsmA, RsmE and RsmI binding to RNA‐CfcR(ab). RNA‐CfcR(ab) includes the predicted Rsm binding motifs A and B (see Supporting Information Fig. S5). Note that specific unlabeled competitor (SUC) RNA‐CfcR(ab) prevented the formation of the labeled RNA‐CfcR(ab)‐protein complexes.

To investigate the potential direct interaction between the Rsm proteins of *P. putida* KT2440 and the motifs matching the consensus for the binding of these proteins found in the leader sequence of the CfcR transcripts, we performed fluorescence‐based electrophoretic mobility shift assays (fEMSA). Three different transcripts RNA‐CfcR(a), RNA‐CfcR(b) and RNA‐CfcR(ab), obtained using *in vitro* transcription, were used in these experiments. The first transcript covered the putative Rsm‐binding motif A, whereas the latter two contained motif B and motifs A and B respectively. While motif B is located in a predicted stem‐heptaloop, an unorthodox Rsm‐binding stem‐loop was predicted around motif A (Supporting Information Fig. S5). Fixed quantities of each of the transcripts labelled with a fluorescent DNA‐probe as described in the ‘Experimental procedures’ section were incubated with increasing concentrations of His‐tagged Rsm proteins (0–1000 nM), and the electrophoretic mobility of the complexes was analyzed in native TBE polyacrylamide gels. The effect of incubating RNA‐CfcR(b) with purified RsmA (100–400 nM), RsmE (400–800 nM) and RsmI (400–800 nM) resulted in RNA‐CfcR(b) shifts when compared with the same experimental condition without protein, indicating their binding to this transcript (Fig. 3). These shifts disappeared when an excess (500 nM) of specific unlabelled competitor (SUC) RNA‐CfcR(b) was added, indicating that RsmA, RsmE and RsmI proteins likely interact with motif B and these interactions are specific (Fig. 3). No interactions were observed between RNA‐CfcR(a) and any of the Rsm proteins (Supporting Information Fig. S6). With RsmA, no obvious shifts in RNA‐CfcR(ab) were observed although by increasing RsmA concentration above 400 and up to 1000 nM the attenuation of a minor band was noticeable and shown to be specific (Fig. 4). Specific RNA‐Cfc(ab) shifts were observed after incubation with RsmE (600–800 nM) and RsmI (200–400 nM) indicating that these Rsm proteins likely interact with motif B encompassed in this transcript. These *in vitro* results strongly suggest that there is a binding motif (motif B) for Rsm proteins at the translation start in the *cfcR* mRNA. Although the binding of RsmA to this motif in RNA‐CfcR(b) transcript showed the highest affinity, the fact that the interaction of this protein with RNA‐Cfc(ab) was not as clear as those of RsmE and RsmI suggests that perhaps the binding between RsmA and the Rsm‐binding motif might be more easily impeded by changes in mRNA secondary structure.

### Motif B overlapping the translation initiation codon in the RNA‐CfcR transcript is essential for the repression of CfcR by Rsm

**Figure 5:**
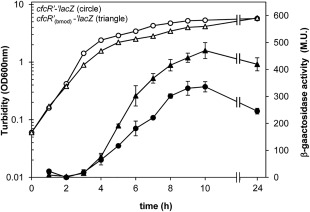
Alteration of Rsm‐binding motif B in RNA‐CfcR affects the expression pattern of *cfcR* in *P. putida* KT2440. Growth (hollow) and β‐galactosidase activity (solid) are plotted for strains with the *cfcR’‐‘lacZ* fusion in pMIR219 (circle) and the *cfcR’*_(bmod)_‐‘*lacZ* fusion in pMIR220 (triangle). Sequence ATGGAT in pMIR219, being ATG the start codon of *cfcR*, was replaced with ATGTTA in pMIR220. Experiments were carried out in triplicate and activities were assayed in duplicate. Average data and standard deviation are plotted from one representative experiment. Statistically significant differences between activities of the fusions were detected from 5 h onwards (Student's *t* test; *P* < 0.05).

**Figure 6:**
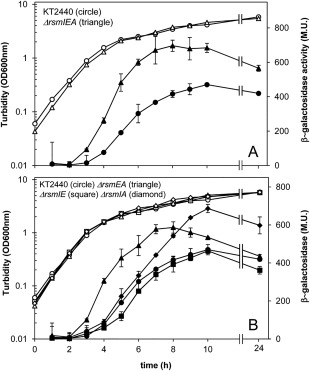
Activity of the translational fusion *cfcR’*_(bmod)_‐*‘lacZ* in *rsm* mutant genetic backgrounds. Cultures growing in LB supplied with Tc as described in the ‘Experimental procedures’ section were analyzed for turbidity (hollow) and β‐galactosidase activities (solid) at the indicated times. A. Data for wild‐type *P. putida* KT2440 (circle) and the *ΔrsmIEA* triple mutant (triangle) strains are shown. B. Data for *P. putida* KT2440 (circle), *ΔrsmEA* (triangle), *ΔrsmIE* (square) and *ΔrsmIA* (diamond) strains are shown. The experiments were performed in triplicate and activities were assayed in duplicate. Average data and standard deviations are plotted from one representative experiment. Statistically significant differences were observed between wild type and *ΔrsmIEA* (from 4 h onwards), *ΔrsmEA* (between 4 and 9 h) and *ΔrsmIA* mutant strains (from 7 h onwards). No differences were observed between wild type and *ΔrsmIE* (Student's *t* test; *P* < 0.05).

The involvement of motif B in the post‐transcriptional repression of *cfcR* was tested *in vivo* using a translational fusion of *cfcR*, containing a modified motif B, to *lacZ* (*cfcR’*_(bmod)_‐*‘lacZ* in pMIR220) (Fig. 1B). The replacement of CATGGATG for CATGTTAG in this fusion destroys the required core ‘GGA’ in the consensus (Dubey *et al*., 2005). The activity of this fusion was compared to that of the fusion with an intact motif B (*cfcR’‐‘lacZ* in pMIR219) in the wild‐type strain. The first construction with the modified motif B reached higher activity levels and its kinetics were very similar to that of *cfcR’‐‘lacZ* in the triple mutant *ΔrsmIEA* (Fig. 5). This result confirmed that motif B is involved in CfcR repression and together with the *in vitro* evidence obtained with fEMSA further supports that Rsm proteins are direct repressors of *cfcR*. The activity of the *cfcR’*_(bmod)_‐*‘lacZ* fusion was also tested in the triple *ΔrsmIEA* mutant and resulted in earlier and higher activity than in the wild type (Fig. 6A) indicating that Rsm‐mediated repression still remained active independently of the modification made to motif B. This fusion with a mutated motif B exhibited similar expression pattern in the three null *rsm* single mutants and the wild type (Supporting Information Fig. S7), and the same result was observed with the double mutant *ΔrsmIE*, where only RsmA is active (Fig. 6B). However, certain derepression was observed in mutants *ΔrsmEA* and *ΔrsmIA* (Fig. 6B). These results indicate that the binding of RsmA was lost after the modification of motif B and suggest together with those obtained in the fEMSA experiments that the repression of *cfcR* exerted at its translation start takes place mainly through RsmA. Certain repressive role of RsmE and RsmI was maintained independently of motif B likely by an indirect way.

### The free c‐di‐GMP pool of *Pseudomonas putida* is negatively regulated by Rsm proteins largely through CfcR

**Figure 7:**
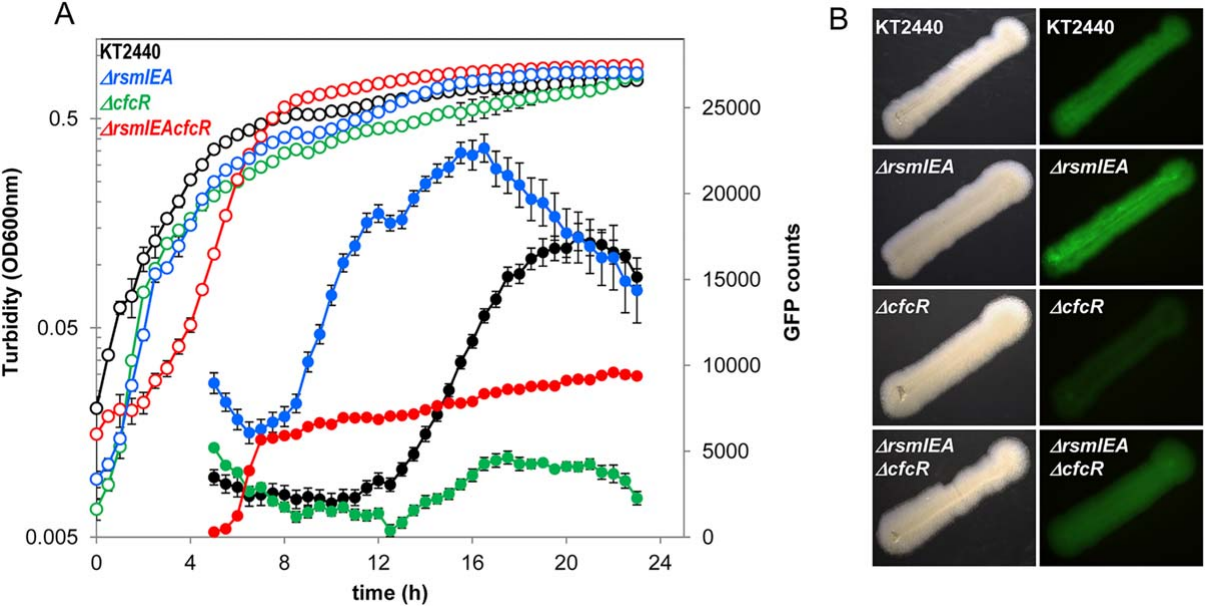
Time course of c‐di‐GMP free pool in *P. putida* strains. All strains harboring the biosensor plasmid pCdrA::*gfp* ^C^. A. Growth (hollow symbols) and GFP counts (solid symbols) indicating fluorescence readings normalized for growth in LB 1/10 (OD_600 nm_). Experiments were carried out in duplicate and measurements were made in triplicate. Average data and standard error is plotted for *Pseudomonas putida* KT2440 (black), *ΔrsmIEA* (blue), *ΔcfcR* (green) and *ΔrsmIEAcfcR* (red) strains. B. LB agar plates were incubated at 30°C for 24 h; pictures of the visible field (left panels) were taken using a Leica stereomicroscope M165FC and for dark field pictures (right panels), an excitation/emission filter 480/510 nm was used with an exposure time of 1.1 s.

**Figure 8:**
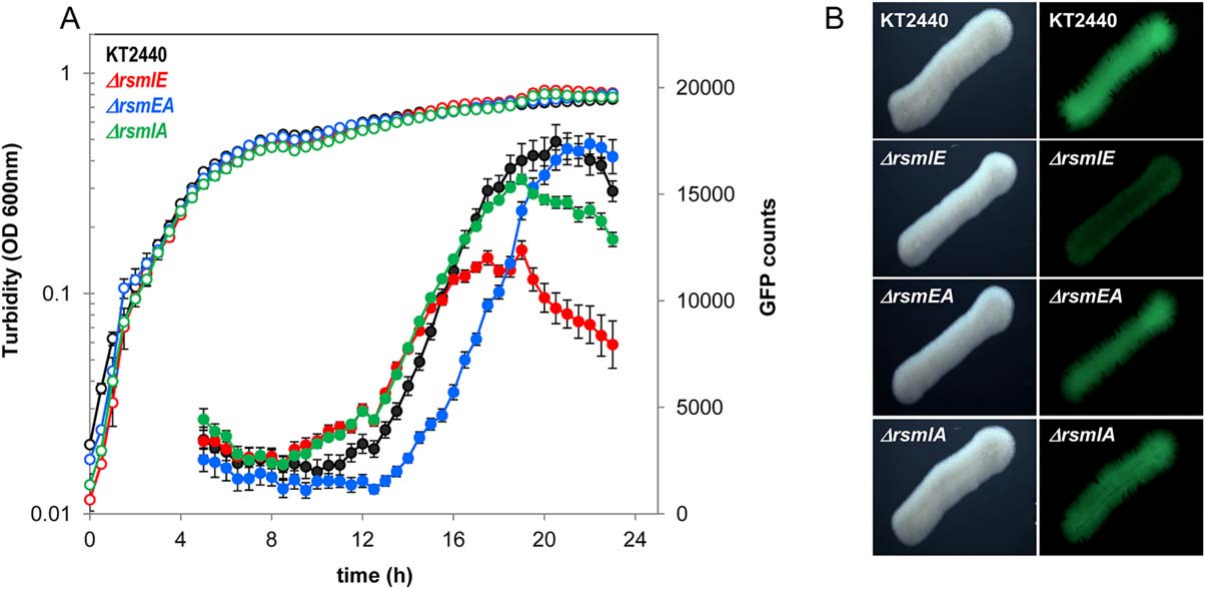
Time course of c‐di‐GMP free pool in double *rsm* mutants. All strains harbour biosensor plasmid pCdrA::gfp^C^. A. Growth (hollow symbols) and GFP counts (solid symbols) that indicate fluorescence readings corrected for growth in LB 1/10 (OD_600 nm_). Experiments were carried out in duplicate with three experimental replicates. Average data and standard error are plotted for *Pseudomonas putida* KT2440 (black), *ΔrsmIE* (red), *ΔrsmEA* (blue) and *ΔrsmIA* (green). B. LB agar plates were incubated at 30°C for 24 h; pictures of the visible field (left panels) were taken using Leica stereomicroscope M165FC and for dark field pictures (right panels), an excitation/emission filter 480/510 nm was used with an exposure time of 1.3 s.

As a consequence of the enhancement of *cfcR* expression in the *ΔrmsIEA* background, we hypothesized that the level of c‐di‐GMP should be increased in this mutant. To investigate this possibility, we monitored c‐di‐GMP levels in the wild‐type strain and compared them to those from the *ΔcfrR*, *ΔrsmIEA* and *ΔrsmIEAcfcR* mutant genetic backgrounds using the biosensor plasmid pCdrA::*gfp^C^* (Rybtke *et al*., 2012). GFP fluorescence was quantified from cultures grown in microtiter plates using an Infinite 200 Tecan fluorimeter and also directly observed from bacterial streaks on agar plates as indicated in the ‘Experimental procedures’ section. In the wild‐type strain, a boost in c‐di‐GMP was observed in the stationary phase of growth, which reached a maximum after 20 h of incubation (Fig. 7A). Interestingly, in the triple *ΔrsmIEA* mutant, the c‐di‐GMP boost was observed earlier (6 h in advance) than in the wild type and although the values at some stages were up to sixfold higher than in the wild type, they were not sustained over time (Fig. 7A). In the *cfcR* mutant, the levels of c‐di‐GMP remained significantly lower than in the wild‐type strain during the stationary phase (Fig. 7A), which was in agreement with *cfcR* being RpoS dependent (Matilla *et al*., 2011). The fact that CfcR was responsible for up to 75% of c‐di‐GMP cell content in this growth stage suggested that the diguanylate cyclase activity of this protein is a major determinant of the free global pool of this second messenger in *P. putida* during the stationary phase. This could be confirmed with the quadruple *ΔrsmIEAcfcR* mutant where the c‐di‐GMP boost in the triple *ΔrsmIEA* genetic background was replaced by a more moderate and earlier increase, at the onset of the stationary phase (Fig. 7A). Visualization by stereomicroscopy of the fluorescence produced by these strains gave results that positively correlated with the quantitative data (Fig. 7B). The kinetics of c‐di‐GMP was also analyzed in the single *rsm* mutants, and no important differences were observed between these strains and the wild type (Supporting Information Fig. S8). Similar kinetics was observed in the double Δ*rsmIA* mutant, with only RsmE active, although with a slight decrease in c‐di‐GMP levels at the end of the experiment (Fig. 8). Different c‐di‐GMP profiles were observed for *ΔrsmEA* and *ΔrsmIE*; in the first strain, with only RsmI active, the boost in the level of the second messenger was delayed in comparison with the wild type, while in the latter, where only RsmA remained active, the maximum values were almost 50% below those attained by the wild type (Fig. 8). This seems to be an indication that the repression exerted by RsmA upon c‐di‐GMP levels (through *cfcR*) is stricter in the absence of RsmE and RsmI and that the last acts later in the stationary phase of growth.

### CfcR is responsible for the enhanced biofilm formation capacity of the triple mutant *ΔrsmIEA*

**Figure 9:**
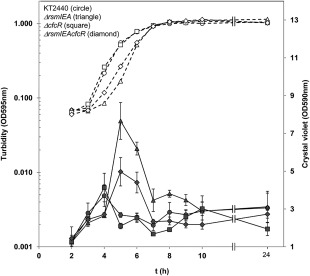
Biofilm formation capacity of *P. putida* strains measured using multiwell plates. Growth (hollow symbols) and biofilm measurements (solid symbols) are shown. Data for wild‐type *P. putida* KT2440 (circle), triple mutant *ΔrsmIEA* (tringle), *ΔcfcR* mutant (square) and quadruple *ΔrsmIEAcfcR* mutant (diamond) strains are shown. The experiment was carried out in quadruplicate. Average data and standard deviations are plotted from a representative experiment using data measured in triplicate. Statistically significant differences were detected at 5 and 6 h between triple and quadruple mutants, between wild type and triple mutants and between wild type and quadruple mutants (Student's *t* test; *P* < 0.05).

It was previously shown that the triple *ΔrsmIEA* mutant formed more biofilm than the wild type when this capacity was analyzed with slight rotation or under static conditions (Huertas‐Rosales *et al*., 2016). In addition, as seen above, the increase in c‐di‐GMP observed in this strain was compromised when a *cfcR* deletion was added to *ΔrmsIEA* mutant. In order to evaluate the contribution of CfcR to the increased biofilm formation capacity observed in the triple *ΔrsmIEA* mutant, we evaluated the biofilm formed by the quadruple mutant *ΔrsmIEAcfcR* in polystyrene multiwell plates under static conditions and found that at the onset of stationary phase, biofilm formation was reduced by half in comparison to the triple mutant (Fig. 9). No difference in biofilm formation capacity between the triple and the quadruple mutant was observed in polycarbonate tubes with optimal aeration (Supporting Information Fig. S9).

## Discussion

In this work, we aimed to unravel the role of the RNA‐binding post‐transcriptional regulators of the CsrA/RsmA family (RsmA, RsmE and RsmI) from *P. putida* (Huertas‐Rosales *et al*., 2016) in the regulation of the diguanylate cyclase CfcR and the impact this control exerts on c‐di‐GMP levels and biofilm formation. We therefore tested if single and combined deletions of the *rsm* genes affected the expression of the translational fusion *cfcR’‐‘lacZ*. Single mutants allowed us to evaluate the effect upon *cfcR* expression of the loss of individual Rsm proteins, while double mutants allowed to investigate the role of each Rsm protein when present in the cells alone (i.e., *ΔrsmIE* for active RsmA; *ΔrsmIA* for active RsmE and *ΔrsmEA* for active RsmI). Expression of *cfcR* was only notably altered (induced) in the triple *ΔrmsIEA* mutant, indicating that Rsm proteins repress *cfcR*, but single mutations may be compensated by the other two Rsm homologues and therefore their roles can be interchangeable. In support of this, we confirmed that the three Rsm proteins could specifically bind the transcript RNA‐CfcR(b) containing motif B, which matches a consensus for Csr/Rsm proteins binding at the start codon of *cfcR*. These *in vitro* results together with the results obtained *in vivo* with the *cfcR’‐‘lacZ* fusion allowed us to confirm the negative regulation of *cfcR* via direct RNA‐binding regulators of the CsrA/RsmA family. Furthermore, the specific role of motif B 5′C**ATG**GATG3′ (start codon in bold) was established after verifying the positive effect of replacing the nucleotides GGA, which are highly conserved (Dubey *et al*., 2005), for GTT in the activity of the fusion c*fcR’*_(bmod)_ *‐‘lacZ* in the wild‐type strain. This confirmed that the repression of *cfcR* by Rsm proteins takes place at the initiation of translation. Given that the activity of this mutated fusion was very slightly reduced in the double mutant *ΔrsmIE*, with an RsmA active, whereas it was enhanced in the other two double mutants with active RsmE or RsmI, we came to conclude that RsmA had a major role in the inhibition of translation initiation, and in fact, it bound with the highest affinity to RNA‐CfcR(b). However, this binding was easily impeded as a consequence of increasing the length of the RNA target likely because of changes provoked in its secondary structure. An heptaloop‐stem is predicted at the binding site in the case of *cfcR* mRNA, instead of the penta‐ or hexaloop more commonly found (Schubert *et al*., 2007; Lapouge *et al*., 2013). Yet to our knowledge, although RsmA structure of *P. putida* has been resolved (Rife *et al*., 2005), a structural model of this protein, or any CsrA/RsmA, contacting target RNA is not available.

We have discarded that Rsm proteins interact with another putative motif (motif A) upstream in the leader of mRNA *cfcR*. In fact, the sequence of this motif A 5′TAATGGATCC3′ differs more than motif B from the consensus 5′^A^/_U_CANGGANG^U^/_A_3′, based on the optimal contacts of the RsmE homodimer with its two RNA‐binding sites (Schubert *et al*., 2007). Thus, in a genetic background with all *rsm* genes deleted, no differences in post‐transcriptional regulation of *cfcR* would be expected from a fusion with an altered motif B. However, increased activity was detected from *cfcR’*_(bmod)_‐*‘lacZ* compared to *cfcR’‐‘lacZ* in the triple mutant *ΔrsmIEA*. This might be explained if the transcription of *cfcR* was activated in this strain. We have confirmed that the expression of RpoS, which was previously shown to positively regulate transcription of *cfcR* (Matilla *et al*., 2011), was anticipated in the *ΔrsmIEA* strain compared to the wild type. This early activation of RpoS could be responsible not only for the enhanced activity of the fusion *cfcR’*_(bmod)_‐*‘lacZ* in the mentioned strains but also contribute to the *cfcR* mRNA level enhancement observed in the double mutants *ΔrsmEA* and *ΔrsmIA* (not in *ΔrsmIE*) and to the shortening in the incubation times necessary to achieve a great boost in the triple mutant. We have identified *rpoS* as a target of Rsm proteins in RIP‐seq experiments (our unpublished results) as an indication that RpoS regulation by Rsm proteins is direct. In *P. protegens* CHA0, RpoS was also found to be negatively regulated by RsmA (Heeb *et al*., 2005). Thus, we can conclude that *cfcR* is negatively regulated by Rsm proteins not only directly at the initiation of its translation but also indirectly at the transcriptional level through RpoS. Nevertheless, it cannot be ruled out that Rsm proteins binding to their target mRNA might alter (shorten) their stability.

We have reported previously that the transcriptional regulators ANR (Matilla *et al*., 2011) and FleQ (Ramos‐González *et al*., 2016) positively modulate the transcription of *cfcR*. Since in the same RlP‐seq experiments, we have identified that ANR is a target of Rsm proteins (our unpublished results), it is tempting to speculate that increased levels of ANR are attained as a consequence of the loss of Rsm proteins. Thus, enhanced ANR levels might also contribute to the changes in the *cfcR* expression observed in the triple *ΔrsmIEA* mutant and to a lesser extent in the double *rsmEA* and *rsmIA* mutants.

Given the low amount of c‐di‐GMP present in *P. putida* KT2440, quantification of the levels of this second messenger in this strain has not been feasible to date using analytical methods. However, identification of mutants with lower levels of c‐di‐GMP has recently been made possible using a c‐di‐GMP biosensor (Ramos‐González *et al*., 2016). In this work, we confirm that c‐di‐GMP values are severely reduced in a *cfcR* mutant during stationary phase, indicating that this DGC is of major relevance to the free pool of c‐di‐GMP in *P. putida* during this growth stage. In addition, the earlier and enhanced expression of *cfcR* observed in the triple mutant *ΔrsmIEA* correlated with an earlier boost of free c‐di‐GMP in this strain, which was compromised when *cfcR* was deleted. While *ΔrsmIEA* had been shown to form more biofilm at earlier stages than wild‐type strain when grown with slight rotation or under static conditions (Huertas‐Rosales *et al*., 2016), in the quadruple mutant *ΔrsmIEAΔcfcR* this increased biofilm formation was also observed although to a lesser extent in static conditions. Therefore, the contribution of CfcR to biofilm formation is more important under conditions with limited aeration – a finding that is in agreement with *cfcR* expression being enhanced under O_2_ depletion (Matilla *et al*., 2011). These observations indicate that out of the 36 genes that have been annotated as encoding GGDEF domain containing proteins in *P. putida* (Ulrich and Zhulin, 2010), *cfcR* is a key player and required for the *ΔrsmIEA* mutant to display both a c‐di‐GMP boost and increased biofilm formation capacity in the conditions tested. C‐di‐GMP values were significantly higher in *ΔrsmIEAcfcR* than in the *cfcR* mutant at earlier stages. This indicates that diguanilate cyclase(s) other than CfcR might also be de‐repressed as a consequence of the loss of Rsm proteins. Nevertheless, c‐di‐GMP levels were maintained during the stationary phase of growth in *ΔrsmIEAΔcfcR* below those observed in the wild type (Fig. 7) indicating that, even if de‐repressed, these putative DGCs likely play only a minor role in the regulation of free c‐di‐GMP during this stage and under the conditions used in our studies. It should be mentioned that other proteins that contribute to regulate c‐di‐GMP levels, such as PDE, might also become differentially regulated as a consequence of *rsm* genes deletion. In *E. coli*, two GGDEF containing proteins (YcdT and YdeH) are post‐transcriptionally regulated by CsrA (Jonas *et al*., 2008). In *P. aeruginosa*, SadC and HsbD are the only DGCs involved in biofilm formation that are controlled by the Gac/Rsm pathway (Moscoso *et al*., 2014; Valentini *et al*., 2016). Interestingly, CfcR has no orthologues in *P. aeruginosa* strains and there are no orthologues of SadC and HsbD in *P. putida* KT2440 (Winsor *et al*., 2016). Thus, our study confirms the link between the Gac/Rsm cascade and c‐di‐GMP signalling, although the proteins involved in each bacterium are different. It should be noticed that the expression of RpoS in *P. putida* requires an active Gac system (Martínez‐Gil *et al*., 2014); therefore, the inactivation of Gac impedes the expression of *cfcR*.

C‐di‐GMP kinetics was similar in the single Rsm mutants and the wild type consistently with the lack of difference found in *cfcR* expression between these strains. When RsmA remained as the only Rsm protein in the double mutant *ΔrsmIE*, it promoted a great decay in the c‐di‐GMP free pool at the stationary phase of growth to the point that values of this second messenger were lower in this strain than in the WT. Since under the conditions tested, most of the c‐di‐GMP free pool of *P. putida* in the stationary phase was due to the presence of CfcR, the low value of this second messenger in the mutant *ΔrsmIE* is in agreement with RsmA being the key inhibitor at the initiation of *cfcR* translation, as it has been mentioned earlier. We hypothetize that RsmA protein levels in the cell are tightly regulated. With three proteins sharing the same RNA target, if RsmA, with the highest affinity, was under‐represented compared to the other two, removing its less efficient competitors RsmE and RsmI, might result in increased repression, which was in fact our observation in the double mutant *ΔrsmIE*. Consistently, RsmE expression levels were higher than those of RsmA (above fivefold), and although the expression of RsmI was inferior to that of RsmA (Huertas‐Rosales *et al*., 2016), an induction on RsmA expression was observed when RsmE and RsmI were deleted. Thus, the co‐existence of three members of the CsrA/RsmA family in *P. putida* seems to allow a finer modulation of *cfcR* regulation especially since self‐regulation and cross‐regulation among the Rsm proteins have been reported (Huertas‐Rosales *et al*., 2016). The phenotype related with a reduced *cfcR* expression in the double mutant having still an RsmA active was noticeable under static conditions (c‐di‐GMP values). However, under shaking with optimal O_2_ availability this effect was not observable, either for second messenger (Supporting Information Fig. S10) or for gene expression values obtained with *cfcR’‐‘lacZ*, which is in agreement with the reduced biofilm formation capacity observed for this mutant specifically in microtiter plates in static (Huertas‐Rosales *et al*., 2016).

**Figure 10:**
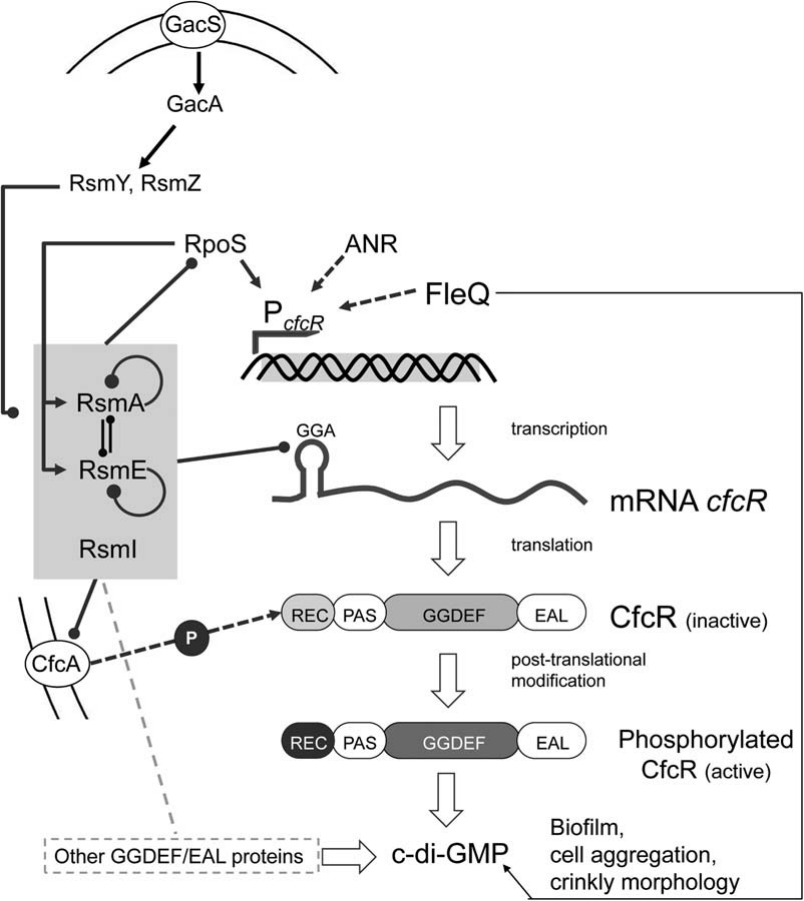
Schematic summary of the regulatory circuits that influence expression and diguanylate cyclase activity of CfcR, ultimately leading to changes in c‐di‐GMP levels. Arrow‐ended and round‐ended lines indicate positive and negative regulation respectively. Broken lines indicate regulatory processes for which evidence exists but need to be studied in further detail. Binding of c‐di‐GMP to FleQ and regulation of Rsm proteins as recently reported by Huertas‐Rosales and colleagues (2016) is included. Rsm proteins regulate the expression of other GGDEF/EAL proteins (Huertas‐Rosales *et al*., in preparation). See text for more details.

The results presented in this work provide evidence that CfcR is a key determinant for the generation of the free pool of c‐di‐GMP in stationary phase in *P. putida*, especially when O_2_ is depleted, and also for the increased biofilm formation observed when Rsm proteins are absent. This central role for CfcR, and the impact that c‐di‐GMP levels have on different phenotypes, may explain why *cfcR* expression is tightly regulated at multiple levels, transcriptionally via RpoS, ANR and FleQ, post‐transcriptionally via direct interaction with Rsm proteins, and post‐translationally via phosphorylation, very likely by the multi‐sensor hybrid histidine kinase CfcA (Fig. 10).

## Experimental procedures

### Bacterial strains, culture media and growth conditions

The bacterial strains and plasmids used in this study are listed in Tables 1 and 2 respectively. *Pseudomonas putida* KT2440 is a plasmid‐free derivative of *P. putida* mt‐2, which was isolated from a vegetable‐planted field and whose genome is sequenced (Nakazawa, 2002; Nelson *et al*., 2002). *Pseudomonas putida* strains were grown at 30°C as indicated, in either Luria‐Bertani (LB) medium (Bertani, 1951) or M9 defined medium (Sambrook *et al*., 1989) supplemented with 1 mM MgSO_4_, 6 mg l^−1^ ammonium ferric citrate and trace metals as described previously (Yousef‐Coronado *et al*., 2008). Glucose (27 mM) or sodium citrate (15 mM) were added as alternative carbon sources to defined M9‐minimal medium. *Escherichia coli* strains were grown at 37°C in LB. When appropriate, antibiotics were added to the medium at the following final concentrations (µg ml^−1^): ampicillin 100; kanamycin 25; streptomycin 50 (*E. coli*) or 100 (*P. putida*); gentamycin 50 or 100 as indicated and tetracycline 10, 20 or 200 as indicated. Cell growth was followed by measuring turbidity at 600 or 660 nm as indicated.

**Table 1:** Bacterial strains used in this work.

Strain	Genotype/relevant characteristics	Reference or source
*Escherichia coli*		
CC118*λpir*	Rif^R^, *λpir*, donor strain for pKNG101‐derivative plasmids	Herrero *et al*., 1990
DH5α	*supE*44 *lac*U169(Ø80*lacZ*ΔM15) *hsdR*17 ( $r_{k}^{-}$ $m_{k}^{-}$) *recA*1 *endA*1 *gyrA*96 *thi*‐1 *relA*1	Woodcock *et al*., 1989
HB101 (pRK600)	Helper strain harboring a Cm^R^ *mob tra* plasmid	V. De Lorenzo
*P. putida*		
KT2440	Wild‐type, prototroph, cured of pWWO derivative of *P. putida* mt‐2	Nakazawa, 2002
*ΔcfcR*	Km^R^, non‐polar null PP_4959 mutant	Matilla *et al*., 2011
*ΔrsmI*	Null PP_1476 derivative of KT2440	Huertas‐Rosales *et al*., 2016
*ΔrsmE*	Null PP_3832 derivative of KT2440	Huertas‐Rosales *et al*., 2016
*ΔrsmA*	Null PP_4472 derivative of KT2440	Huertas‐Rosales *et al*., 2016
*ΔrsmIE*	Double null PP_1746/PP_3832 derivative of KT2440	Huertas‐Rosales *et al*., 2016
*ΔrsmEA*	Double null PP_3832/PP_4472 derivative of KT2440	Huertas‐Rosales *et al*., 2016
*ΔrsmIA*	Double null PP_1746/PP_4472 derivative of KT2440	Huertas‐Rosales *et al*., 2016
*ΔrsmIEA*	Triple null PP_1746/PP_3832/PP_4472 derivative of KT2440	Huertas‐Rosales *et al*., 2016
*ΔrsmIEAcfcR*	PP_4959 null derivative of *ΔrsmIEA*	This study

Cm, chloramphenicol; Gm, gentamicin; Km, kanamycin; Sm, streptomycin; Tc, tetracycline.

**Table 2:** Plasmids used in this work.

Plasmid	Relevant characteristics	Reference or source
pBBR1‐MCS5	Gm^r^, *oriRK2 mobRK2*	Kovach *et al*., 1995
pCdrA::*gfp* ^C^	Ap^r^, Gm^R^, FleQ dependent c‐di‐GMP biosensor	Rybtke *et al*., 2012
pCR^TM^2.1‐TOPO	Km^R^, PCR cloning vector with β‐galactosidase α‐complementation	Invitrogen, Waltham, MA
pGEM®‐T	Ap^R^, PCR cloning vector with β‐galactosidase α‐complementation	Promega
pKNG101	Sm^R^, *oriR6K mobRK2 sacBR*	Kaniga *et al*., 1991
pMP220	Tc^R^, oriRK2, *‘lacZ*, vector used for transcriptional fusions	Spaink *et al*., 1987
pMP220‐BamHI	Tc^R^, derivative of pMP220 with a deletion of a 238‐bp BamHI fragment, which removes the ribosome binding site and 52 codons of the *cat* gene that precede ‘*lacZ;* used as vector for translational fusions	Matilla *et al*., 2011
pUC18Not	Ap^r^, derivative of pUC18 with two NotI sites flanking the MCS	Herrero *et al*., 1990
pMAMV21	Tc^R^, *rpoS’‐‘lacZ* translational fusion in pMP220‐BamHI	Matilla *et al*., 2011
pME6032	Tc^R^, pVS1‐p15A derivative. Broad host range *lacI^*q*^*‐P*_*tac*_* expression vector	Heeb *et al*., 2002
pME6032‐*rsmA*	Tc^R^; pME6032 derivative for the ectopic expression of *rsmA* under the control of *lacl^*q*^*‐P*_*tac*_*	Huertas‐Rosales *et al*., 2016
pME6032‐*rsmE*	Tc^R^; pME6032 derivative for the ectopic expression of *rsmE* under the control of *lacl^*q*^*‐P*_*tac*_*	Huertas‐Rosales *et al*., 2016
pME6032‐*rsmI*	Tc^R^; pME6032 derivative for the ectopic expression of *rsmI* under the control of *lacl^*q*^*‐P*_*tac*_*	Huertas‐Rosales *et al*., 2016
pMIR153	Km^R^, pKNG101 derivative harboring *cfcR* inactivation	Matilla *et al*., 2011
pMIR219	Tc^R^, *cfcR’‐‘lacZ* translational fusion in pMP220‐BamHI	This study
pMIR200	Tc^R^, *cfcR’::‘lacZ* transcriptional fusion in pMP220	This study
pMIR220	Tc^R^, *cfcR’*_(bmod)_‐‘*lacZ* translational fusion in pMP220‐BamHI	This study

Ap, ampicillin; Cm, chloramphenicol; Gm, gentamicin; Km, kanamycin; MCS, multicloning site; Tc, tetracycline.

### DNA techniques

Digestion with restriction enzymes, dephosphorylation, ligation and electrophoresis were carried out using standard methods (Ausubel *et al*, 1987; Sambrook *et al*., 1989), following the manufacturers’ instructions. Plasmid DNA isolation and recovery of DNA fragments from agarose gels were done using Qiagen (Venlo, Netherlands) miniprep and gel extraction kits respectively. Competent cells were prepared using calcium chloride and transformations were performed using standard protocols (Sambrook *et al*., 1989). Electrotransformation of freshly plated *Pseudomonas* cells was performed as previously described (Enderle and Farwell, 1998). Polymerase chain reactions (PCR) were carried out using Taq DNA polymerase (Roche, Basel, Switzerland).

### Triparental conjugations

Transfer of plasmids from *E. coli* to *P. putida* strains was performed by triparental matings using *E. coli* (pRK600) as a helper. For each strain, cells were collected from 0.5 ml of overnight LB cultures via centrifugation, then rinsed and suspended in 50 µl of fresh LB, and finally spotted on mating filter (0.25 µm pore diameter) on LB agar plates. After overnight incubation at 30°C, cells were scraped off from the mating filter and suspended in 2 ml of M9 salts media and serial dilutions were plated on selective citrate‐supplied M9 minimal medium with the appropriate antibiotics to select exconjugants and counterselect donor, helper and recipient strains.

### RNA purification

*Pseudomonas putida* bacterial cells were incubated at 30°C with orbital shaking (200 rpm), and samples harvested at indicated times by centrifugation, instantly frozen with liquid nitrogen and stored at −80°C. Total RNA was extracted using an RNA isolation kit (Macherey‐Nagel, Düren, Germany), following the manufacturer's instructions. RNA samples were subsequently treated with Turbo DNA‐free kit (Ambion, Foster City, CA) to remove DNA traces, as specified by the supplier. RNA concentration was determined using the NanoDrop ND1000 spectrophotometer (NanoDrop Technologies, Inc., Wilmington, DE, USA), RNA integrity was assessed by agarose gel electrophoresis, and the absence of DNA was verified by PCR.

### Quantitative real‐time PCR *(qRT‐PCR)*

Analysis by qRT‐PCR was performed using total RNA preparations obtained from three independent cultures (three biological replicates) using iCycler Iq (Bio‐Rad, Hercules, CA, USA). DNA‐free RNA samples (1 µg) were retrotranscribed to cDNA using Superscript II reverse transcriptase (Invitrogen, Waltham, MA) and random hexamers as primers. Template cDNA from the experimental and reference samples was amplified using the primers listed in Supporting Information Table S1. Three experimental replicates were amplified. Each reaction contained 2 µl of a dilution of the target cDNA (1:10–1:10,000) and 23 µl SyBR Green mix (Molecular Probes, Eugene, OR). Samples were initially denatured by heating at 95°C for 10 min. A 40‐cycle amplification and quantification program was then followed (95°C for 15 s, 62°C for 30 s and 72°C for 20 s) with a single fluorescence measurement per cycle according to manufacturer's recommendations. PCR products were between 150 and 200 bp in length. To confirm the amplification of a single PCR product, a melting curve was obtained by slow heating from 60 to 99.5°C at a rate of 0.5°C every 10 s, for 80 cycles, with continuous fluorescence scanning. The results were analysed by means of the comparative critical threshold (ΔΔCt) method (Pfaffl, 2001) and normalized to those obtained for 16S rRNA.

### Generation of *ΔIEAcfcR* mutant by homologous recombination

The pMIR153 plasmid (a derivative of pKNG101) containing the inactivated *cfcR* allele (Matilla *et al*., 2011) was mobilized from *E. coli* CC118 *λpir* into *P. putida ΔrsmIEA* (Huertas‐Rosales *et al*., 2016) by conjugation using HB101 (pRK600) as a helper, as described above. Merodiploid exconjugants were first selected in minimal medium with citrate and streptomycin and then incubated in LB medium supplied with 12% sucrose to obtain clones in which a second recombination event had removed the plasmid backbone. Sm‐sensitive clones were re‐isolated and the presence of the *cfcR* mutation was checked by PCR, followed by sequencing of the corresponding chromosomal region and Southern blotting.

### Construction of *cfcR’‐'lacZ* translational fusions and P_*cfcR*_ *::'lacZ* transcriptional fusion

Two translational fusions, one containing a native motif B and the other containing a modified motif B, were designed to ensure in‐frame cloning to ‘*lacZ* in pMP220‐BamHI (Table 2). PCR amplicons of 261 bp contained the *cfcR* promoter, both +1 sites previously determined experimentally (Matilla *et al*., 2011), ribosome binding site (RBS) and the first 13 nucleotides of the gene. Primers used are listed in Supporting Information Table S1. These amplicons were cloned into pCR2.1‐TOPO to generate pMIR217 and pMIR218, respectively. The absence of mutations was assessed by sequencing. Subsequently, these plasmids were double digested with Acc65I/BglII and the resulting fragments were each cloned into Acc65I/BamHI sites of pMP220‐BamHI to yield pMIR219 and pMIR220.

The transcriptional fusion expanded 246 bp that contained the *cfcR* promoter and both +1 sites of the gene. Primers used are listed in Supporting Information Table S1. This amplicon had been cloned into pGEM®‐T to generate pMIR199. The absence of mutations was assessed by sequencing. Subsequently, these plasmids were double digested with Acc65I/SphI, and the resulting fragment was cloned in pMP220 to yield pMIR200. RBS and ATG for *‘lacZ* in pMIR200 were those of *cat* gene in pMP220.

### Assay for β‐galactosidase activity

Specific β‐galactosidase activity from bacterial suspensions growing in liquid cultures was measured as described (Miller, 1972). An overnight culture of the strain of interest was diluted 1/100 in fresh LB medium supplied with the required antibiotics and grown at 30°C for 1 h. Then the cultures where diluted 1/2 in fresh LB medium and grown at 30°C for 1 more hour to better dilute out any remaining β‐galactosidase that may have accumulated in overnight cultures. Finally cultures were diluted to an OD_600 nm_ of 0.05 (time 0). Cells were then incubated at 30°C under orbital shaking (200 rpm). At the indicated time points, aliquots were measured for optical density (*A*_600_) and β‐galactosidase activity. Experiments were carried out in triplicate on two experimental replicates.

### Production of Rsm proteins

The expression plasmid pME6032 (Heeb *et al*., 2002) was used to express His‐tagged Rsm proteins (His6‐Rsm) in their natural host *P. putida* KT2440. Overnight cultures (10 ml) of KT2440 harbouring plasmids for His6‐Rsm expression, pME6032‐*rsmA*, pME6032‐*rsmE* and pME6032‐*rsmI* (Huertas‐Rosales *et al*., 2016) were used to inoculate LB rich medium (500 ml) containing the appropriate antibiotic. The later cultures were incubated at 30°C with shaking to reach an OD_660 nm_ of 0.8. At this point, expression of His6‐Rsm was induced by the addition of IPTG to a final concentration of 0.5 mM. After 6 h, bacterial cells were harvested by centrifugation and cell pellets stored at −80°C. His6‐Rsm was purified using Ni‐NTA Fast Start Kit (Qiagen, Venlo, Netherlands). Purifications from cultures involving the empty vector plasmid pME6032 were performed and used as controls for any unspecific binding in EMSA. Because proteins were not purified to homogeneity, the level of purity observed in gels was taken into account in the final quantification. Bradford assay and spectrophotometry techniques were used for protein quantification.

### RNA synthesis and fluorescence‐based electrophoretic mobility shift assays (fEMSA)

DNA‐CfcR(ab), DNA‐CfcR(a) and DNA‐Cfc(b) templates were generated by PCR using primers that incorporated a T7 promoter at the 5′ end and a 17 nt tag at the 3′ end (Supporting Information Table S1). These PCR amplicons were then used for the synthesis of RNA probes using MAXIscript T7 kit (Life Technologies). RNA molecules thus obtained, and free of DNA could be detected by hybridization with an ATTO700‐labelled primer as described by Ying and colleagues (2007). The concentration of this primer in the hybridization reaction was in excess (20‐fold >RNA concentration) in order to maximize RNA detection. Purified His6‐Rsm proteins at the indicated concentrations were incubated with 5 nM RNA probe in 1× binding buffer (10 mM Tris‐Cl pH [7.5], 10 mM MgCl_2_, 100 mM KCl), 0.5 µg µl^−1^ total yeast tRNA (Life Technologies), 7.5% (vol/vol) glycerol, 0.2 units SUPERase In RNase Inhibitor (Life Technologies, Carlsbard, CA). Reactions with or without unlabelled competitor RNA (500 nM) were incubated for 30 min at 30°C, then bromophenol blue was added (0.01%, wt/vol), and immediately, the samples were subjected to electrophoresis at 4°C on 6% (wt/vol) native polyacrylamide TBE gel (47 mM Tris, 45 mM boric acid, 1 mM EDTA, pH [8.3]). Images were obtained using a 9201 Odyssey Imaging System (LI‐COR Biosciences, Lincoln, NE) with Image Studio V5.0 software.

### Microtiter plate‐based c‐di‐GMP reporter assays

Microtiter plate‐based assays containing the pCdrA::*gfp^C^* reporter strains were carried out as follows. LB overnight cultures were diluted to an OD_600 nm_ of 0.05 in 1/10 LB in the presence of 20 µg ml^−1^ Gm. Growth (OD_600 nm_) and fluorescence (excitation/reading filter 485/535 nm) were monitored in an Infinite 200 Tecan plate reader using Greiner 96 well plates (black flat bottom polystyrene wells). The assays were conducted in triplicate for 24 h in static with a pulse of shaking just before the measures were registered every 30 min. Optical density values of LB and fluorescence values of KT2440 without reporter plasmid pCdrA::*gfp^C^* were subtracted from all readings (turbidity and fluorescence respectively).

### Biofilm assays

Time course biofilm formation was assayed by determining the ability of cells to grow adhered to the wells of sterile polystyrene microtiter plates (96 flat base multiwell) as previously described (O'Toole and Kolter, 1998) and monitored in a Tecan Sunrise plate reader. An overnight LB culture was diluted down to a final OD_660 nm_ of 0.05 in fresh medium and dispensed at 200 µl per well. Inoculated plates were incubated under static conditions at 30°C for up to 24 h. In order to measure the degree of attachment, every 2 h culture was removed from selected wells, which were then rinsed with 200 µl of distilled water and processed with crystal violet, as detailed later. In addition, biofilm formation was measured in borosilicate tubes. Initially, overnight LB cultures were diluted to an OD_660 nm_ of 0.05 in fresh medium and 2 ml of this cell suspension was added to 16 mm × 150 mm borosilicate glass tubes, which were incubated in a tube rotator at 40 rpm for up to 24 h. At indicated points, non‐adhered cells were removed and the biofilms rinsed with distilled water. Biofilms were stained by the addition of 200 µl (or 5 ml to the tubes) of 1% crystal violet (Sigma, St. Louis, MO) for 15 min followed by rinsing with distilled water. Photos were taken and the cell‐associated dye was solubilized in 200 µl of acetic acid 30% (vol/vol) and quantified by measuring the OD_590 nm_ of the resulting solution. Experiments were performed twice in triplicate.

### Microscopy

Images were taken using Leica stereomicroscope M165FC. Excitation/emission filter 480/510 nm was used for monitoring GFP fluorescence. Required exposure times varied as indicated.

### Statistical methods

Student's *t*‐test for independent samples (*P* < 0.01 or *P* < 0.05) was applied as appropriate using ‘R’ program for all statistical analyses.

## Supporting information

Additional Supporting Information may be found in the online version of this article at the publisher's web‐site:

**Table S1.** Primers used in this work.
**Fig. S1.** Activity of the *cfcR’‐‘lacZ* fusion in single and double *rsm* mutants. LB cultures, prepared as described in the ‘Experimental procedures’ section, were analyzed for turbidity (hollow symbols) and β‐galactosidase activity (solid symbols) at the indicated times. A. wild‐type KT2440 (circle), *rsmA* (triangle), *rsmE* (square) and *rsmI* (diamond); B. wild‐type KT2440 (circle), *rsmEA* (triangle), *rsmIE* (square) and *rsmIA* (diamond). Experiments were performed in triplicate. Average and standard deviations are plotted for one representative experiment using data obtained from activities measured in duplicate. Statistically significant differences in β‐galactosidase values were detected between wild type and *ΔrsmI* (at 8 h); *ΔrsmEA* (at 5, 6, 7 and 24 h); *ΔrsmIA* (at 5, 6 and 7 h) and *ΔrsmIE* (at 5 h) (Student's *t* test; *P* < 0.05). Non‐significant differences are not pointed out.
**Fig. S2.** Time course of the relative quantities of *cfcR* mRNA in the single *rsm* mutants versus the wild‐type *P. putida* KT2440 strain. Fold changes were calculated using qRT‐PCR data. Averages and standard deviation of three biological replicates and three experimental replicates are plotted. Asterisks indicate when values for each mutant are significantly different from wild‐type values (Student's *t* test, *P* < 0.05). Fold change of 2 is indicated with a dotted line.
**Fig. S3.** Time course of the relative quantities of *cfcR* mRNA in the double *rsm* mutants versus the wild‐type *P. putida* KT2440 strain. Fold changes were calculated using qRT‐PCR data. Averages and standard deviation of three biological replicates and three experimental replicates are plotted. Asterisks indicate when values for each mutant are significantly different from wild‐type values (Student's *t* test, *P* < 0.05). Fold change of 2 is indicated with a dotted line.
**Fig. S4.** RpoS expression in *P. putida* KT2440 (circle) and triple mutant *ΔrsmIEA* (triangle) strains. Top: activity of the translational *rpoS’‐‘lacZ* fusion. LB cultures supplied with Tc were obtained as indicated in the ‘Experimental procedures’ section. Samples were analyzed for turbidity (hollow symbols) and β‐galactosidase activity (solid symbols) at the indicated times. Experiments were performed in six biological replicates. Average and standard deviation of data from one representative experiment with two experimental replicates are plotted. Statistically significant differences in β‐galactosidase values at 4.5 h were detected in every biological replicate (Student's *t* test; *P* < 0.05). Bottom: time course of the relative quantities of *rpoS* mRNA in the triple mutant *ΔrsmIEA* versus the wild‐type *P. putida* KT2440 strain. Fold changes were calculated using qRT‐PCR data. Averages and standard deviation of three biological replicates and three experimental replicates are plotted.
**Fig. S5.** RNA‐CfcR transcripts used in fEMSA. All transcripts contain two tags that are not included in the sequences shown, one in their 5′ ends with the sequence for the T7 polymerase promoter (5′UUUUCUGCAGUAAUACGACUCACUAUAGG3′) and another in their 3′ ends with the sequence (5′UUUUUUUUGGGGGGGGG3′) complementary to the DNA probe labelled with ATTO 700 fluorescent dye (see ‘Experimental procedures’ section). Nucleotides matching the consensus for Rsm binding in motifs A and B are boxed. The translation start codon of *cfcR* is in bold. Coding sequence of *cfcR* is in italic. Secondary structure predictions of RNAs obtained at mfold web server (Zuker, 2003) are shown. Motif A and motif B are indicated by arrows black and red respectively. Maximum distance between paired bases was established in 30.
**Fig. S6.** Fluorescence‐based EMSA of RsmA, RsmE and RsmI proteins binding to RNA‐CfcR(a). RNA‐CfcR(a) spans more nucleotides than the predicted Rsm binding motif A (see Fig. S5). Note that specific unlabeled competitor SUC RNA‐CfcR(a) did not prevent the formation of the labeled RNA‐CfcR(a)‐RsmE complexes, indicating non‐specific binding.
**Fig. S7.** Activity of the translational fusion *cfcR*_(bmod)_ *’‐‘lacZ* in single *rsm* mutant strains. LB cultures supplied with Tc were obtained as indicated in the ‘Experimental procedures’ section. Samples were analyzed for turbidity (hollow symbols) and B‐galactosidase activity (solid symbols) at the indicated times. Wild‐type KT2440 (circle), *ΔrsmE* (triangle), *ΔrsmI* (square) and *ΔrsmA* (diamond). Experiments were performed in triplicate. Average and standard deviation of data from one representative experiment with two experimental replicates are plotted.
**Fig. S8.** Modulation of c‐di‐GMP cell content by Rsm proteins in single *rsm* mutants. All strains harbour biosensor plasmid pCdrA::*gfp^*C*^*. A. Growth (hollow symbols) and GFP counts (solid symbols) that indicate fluorescence readings corrected for growth in LB 1/10 (OD_600 nm_). Experiments were carried out in duplicate with three experimental replicates. Average data and standard error are plotted for *Pseudomonas putida* KT2440 (circles), *ΔrsmI* (triangle), *ΔrsmE* (square) and *ΔrsmA* (diamond). B. LB agar plates were incubated at 30°C for 24 h; pictures of the visible field (left panels) were taken using Leica stereomicroscope M165FC and for dark field pictures (right panels), an excitation/emission filter 480/510 nm was used with an exposure time of 1.3 s.
**Fig. S9.** Time course of biofilm formation capacity in wt, triple null *rsm* and quadruple *ΔrsmIEAcfcR* mutant strains in borosilicate glass tubes. Experiments were performed as indicated in the ‘Experimental procedures’ section and photos were taken at the indicated times.
**Fig. S10**. Time course of c‐di‐GMP free pool of *P. putida* KT2440 (pCdrA) and *ΔrsmIE* (pCdrA) strains under shaking. LB cultures of KT2440 (pCdrA) (grey) and *ΔrsmIE* (pCdrA) (white) supplied with Gm50 and Pip30 were incubated in flasks under shaking (200 rpm) and diluted at the indicated times at an OD_660 nm_ = 0.15 before measuring fluorescence in a LPS‐220B fluorometer (Photon Technology International) with *λ*_ex_ 485 nm and *λ*_em_ 510 nm. Average and standard deviation of data from two experiments with two experimental replicates are plotted. Cultures without pCdrA exhibited a background of 145 ± 3 (1000×). Statistically significant differences in fluorescence were detected between wild type and *ΔrsmIE* at 7 h (Student's *t* test; *P* < 0.05).
