# Supplementary material for: The Pseudomonas putida CsrA/RsmA homologues negatively affect c‐di‐GMP pools and biofilm formation through the GGDEF/EAL response regulator CfcR
Source: Environ Microbiol. 2017 Jul 21;19(9):3551–66. doi: 10.1111/1462-2920.13848 (PMC6849547; doi:10.1111/1462-2920.13848)
Supplement: Supplementary file 1 — Table S1. Primers used in this work. Fig. S1. Activity of the cfcR’‐‘lacZ fusion in single and double rsm mutants. LB cultures, prepared as described in the ‘Experimental procedures’ section, were analyzed for turbidity (hollow symbols) and β‐galactosidase activity (solid symbols) at the indicated times. A. wild‐type KT2440 (circle), rsmA (triangle), rsmE (square) and rsmI (diamond); B. wild‐type KT2440 (circle), rsmEA (triangle), rsmIE (square) and rsmIA (diamond). Experiments were performed in triplicate. Average and standard deviations are plotted for one representative experiment using data obtained from activities measured in duplicate. Statistically significant differences in β‐galactosidase values were detected between wild type and ΔrsmI (at 8 h); ΔrsmEA (at 5, 6, 7 and 24 h); ΔrsmIA (at 5, 6 and 7 h) and ΔrsmIE (at 5 h) (Student's t test; P < 0.05). Non‐significant differences are not pointed out. Fig. S2. Time course of the relative quantities of cfcR mRNA in the single rsm mutants versus the wild‐type P. putida KT2440 strain. Fold changes were calculated using qRT‐PCR data. Averages and standard deviation of three biological replicates and three experimental replicates are plotted. Asterisks indicate when values for each mutant are significantly different from wild‐type values (Student's t test, P < 0.05). Fold change of 2 is indicated with a dotted line. Fig. S3. Time course of the relative quantities of cfcR mRNA in the double rsm mutants versus the wild‐type P. putida KT2440 strain. Fold changes were calculated using qRT‐PCR data. Averages and standard deviation of three biological replicates and three experimental replicates are plotted. Asterisks indicate when values for each mutant are significantly different from wild‐type values (Student's t test, P < 0.05). Fold change of 2 is indicated with a dotted line. Fig. S4. RpoS expression in P. putida KT2440 (circle) and triple mutant ΔrsmIEA (triangle) strains. Top: activity of the translational rpoS’‐‘ [file EMI-19-3551-s001.docx]

**Suporting information**

**Fig. S1.** Activity of the *cfcR’-‘lacZ* fusion in single and double *rsm* mutants.

**Fig. S2.** Time course of the relative quantities of CfcR-RNA in the single *rsm* mutants versus the wild type *P. putida* KT2440 strain.

**Fig. S3.** Time course of the relative quantities of CfcR-RNA in the double *rsm* mutants versus the wild type *P. putida* KT2440 strain.

**Fig. S4.** RpoS expression in *P. putida* KT2440 (circle) and triple mutant *ΔrsmIEA* (triangle) strains.

**Fig. S5.** RNA-CfcR transcripts used in fEMSA.

**Fig. S6.** Fluorescence-based EMSA of RsmE, RsmI and RsmA proteins binding to RNA-CfcR(a).

**Fig. S7.** Activity of the translational fusion *cfcR*_(bmod)_*’-‘lacZ* in single *rsm* mutant strains.

**Fig. S8.** Modulation of c-di-GMP cell content by Rsm proteins in single *rsm* mutants.

**Fig. S9.** Time course of biofilm formation capacity of wt, triple null *rsm* and quadruple *ΔrsmIEAcfcR* mutant strains in borisilicate glass tubes*.*

**Fig. S10.** Time course of c-di-GMP free pool of *P. putida* KT2440 (pCdrA) and *ΔrsmIE* (pCdrA) strains under shaking.

**Table S1.** Primers used in this work.

**References**

**Fig. S1.** Activity of the *cfcR’-‘lacZ* fusion in single and double *rsm* mutants. LB cultures, prepared as described in the experimental procedures section, were analyzed for turbidity (hollow symbols) and β-galactosidase activity (solid symbols) at the indicated times. A, wild type KT2440 (circle), *rsmA* (triangle), *rsmE* (square) and *rsmI* (diamond); B, wild type KT2440 (circle), *rsmEA* (triangle), *rsmIE* (square) and *rsmIA* (diamond). Experiments were performed in triplicate. Average and standard deviations are plotted for one representative experiment using data obtained from activities measured in duplicate. Statistically significant differences in β-galactosidase values were detected between wild type and *ΔrsmI* (at 8 h); *ΔrsmEA* (at 5, 6 and 7, and 24 h); *ΔrsmIA* (at 5, 6 and 7 h); and *ΔrsmIE* (at 5 h) (Student’s *t* test; *P*< 0.05). Non significant differences are not pointed out.

**Fig. S2.** Time course of the relative quantities of CfcR-RNA in the single *rsm* mutants versus the wild type *P. putida* KT2440 strain. Fold changes were calculated using qRT-PCR data. Averages and standard deviation of three biological replicates and three experimental replicates are plotted. Asterisks indicate when values for each mutant are significantly different from wild type values (Student’s t test, *p*<0.05). Fold change of 2 is indicated with a dotted line.

**Fig. S3.** Time course of the relative quantities of CfcR-RNA in the double *rsm* mutants versus the wild type *P. putida* KT2440 strain. Fold changes were calculated using qRT-PCR data. Averages and standard deviation of three biological replicates and three experimental replicates are plotted. Asterisks indicate when values for each mutant are significantly different from wild-type values (Student’s t test, *p*<0.05). Fold change of 2 is indicated with a dotted line.

**Fig. S4.** RpoS expression in *P. putida* KT2440 (circle) and triple mutant *ΔrsmIEA* (triangle) strains. Top: Activity of the translational *rpoS’-‘lacZ* fusion. LB cultures supplied with Tc were obtained as indicated in the experimental procedures section. Samples were analyzed for turbidity (hollow symbols) and β-galactosidase activity (solid symbols) at the indicated times. Experiments were performed in six biological replicates. Average and standard deviation of data from one representative experiment with two experimental replicates are plotted. Statistically significant differences in β-galactosidase values at 4.5 h were detected in every biological replicate (Student’s *t* test; *P*< 0.05). Bottom: Time course of the relative quantities of RpoS-RNA in the triple mutant *ΔrsmIEA* versus the wild type *P. putida* KT2440 strain. Fold changes were calculated using qRT-PCR data. Averages and standard deviation of three biological replicates and three experimental replicates are plotted.

**
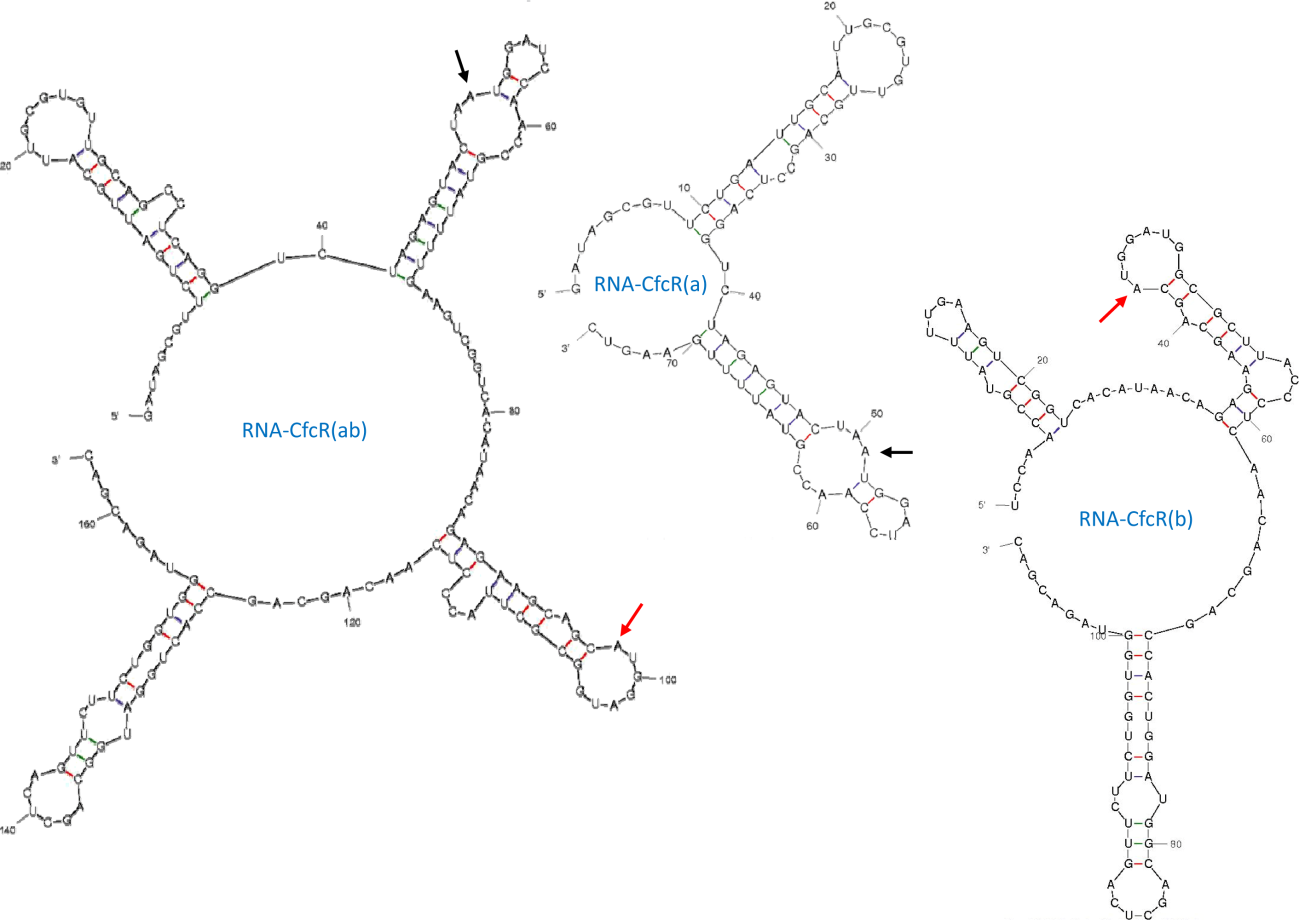
**

**Fig. S5.** RNA-CfcR transcripts used in fEMSA. All transcripts contain two tags that are not included in the sequences shown, one in their 5’ ends with the sequence for the T7 polymerase promoter (5’UUUUCUGCAGUAAUACGACUCACUAUAGG3’), and another in their 3’ ends with the sequence (5’UUUUUUUUGGGGGGGGG3’) complementary to the DNA probe labeled with ATTO 700 fluorescent dye (See Materials and methods section). Nucleotides matching the consensus for Rsm binding in motifs A and B are boxed. The translation start codon of *cfcR* is in bold. Secondary structure predictions of RNAs obtained at m-fold web server (Zucker, 2003) are shown. Motif A and motif B are indicated by arrows black and red, respectively. Maximum distance between paired bases was established in 30.

**Fig. S6.** Fluorescence-based EMSA of RsmE, RsmI and RsmA proteins binding to RNA-CfcR(a). RNA-CfcR(a) spans more nucleotides than the predicted Rsm binding motif A (see Figure 4). Note that specific unlabeled competitor SUC RNA-CfcR(a) did not prevent the formation of the labeled RNA-CfcR(a)-RsmE complexes, indicating non-specific binding.

**Fig. S7.** Activity of the translational fusion *cfcR*_(bmod)_*’-‘lacZ* in single *rsm* mutant strains. LB cultures supplied with Tc were obtained as indicated in the Experimental procedures section. Samples were analyzed for turbidity (hollow symbols) and B-galactosidase activity (solid symbols) at the indicated times. Wild type KT2440 (circle), *ΔrsmE* (triangle), *ΔrsmI* (square) and *ΔrsmA* (diamond). Experiments were performed in triplicate. Average and standard deviation of data from one representative experiment with two experimental replicates are plotted.

**Fig. S8.** Modulation of c-di-GMP cell content by Rsm proteins in single *rsm* mutants. All strains harbor biosensor plasmid pCdrA::*gfp^C^*. A, Growth (hollow symbols) and GFP counts (solid symbols) that indicate fluorescence readings corrected for growth in LB 1/10 (OD_600nm_). Experiments were carried out in duplicate with three experimental replicates. Average data and standard error are plotted for *Pseudomonas putida* KT2440 (circles), *ΔrsmI* (triangle), *ΔrsmE* (square) and *ΔrsmA* (diamond). B, LB-agar plates were incubated at 30°C for 24 h; pictures of the visible field (left panels) were taken using Leica stereomicroscope M165FC and for dark field pictures (right panels), an excitation/emission filter 480/510 nm was used with an exposure time of 1.3 seconds.


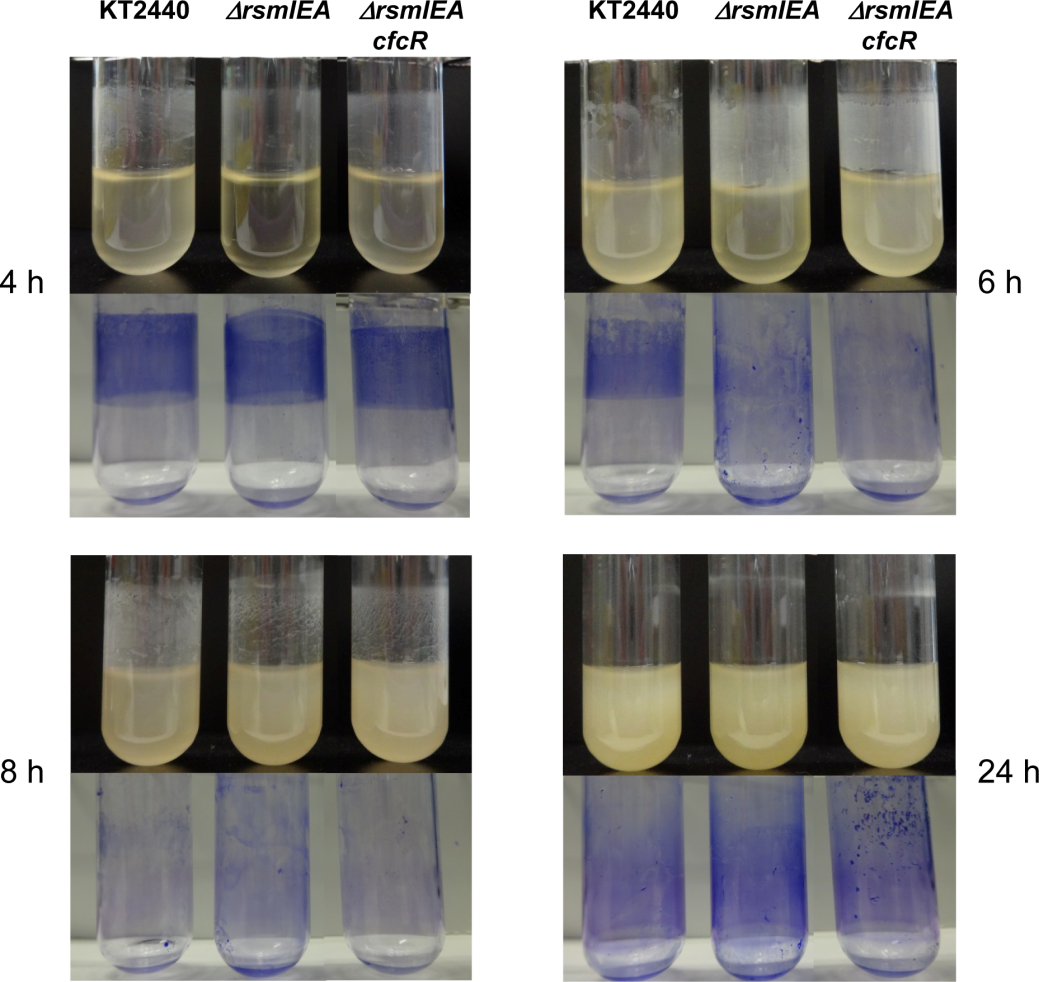


**Fig. S9.** Time course of biofilm formation capacity in wt, triple null *rsm* and quadruple *ΔrsmIEAcfcR* mutant strains in borosilicate glass tubes*.* Experiments were performed as indicated in the experimental procedures and photos were taken at the indicated times.

**Fig. S10**. Time course of c-di-GMP free pool of *P. putida* KT2440 (pCdrA) and *ΔrsmIE* (pCdrA) strains under shaking. LB cultures of KT2440 (pCdrA) (grey) and *ΔrsmIE* (pCdrA) (white) supplied with Gm50 and Pip30 were incubated in flasks under shaking (200 r.p.m) and diluted at the indicated times at an OD_660nm_= 0.15 before measuring fluorescence in a LPS-220B fluorometer (Photon Technology International) with λ_ex_ 485 nm and λ_em_ 510 nm. Average and standard deviation of data from two experiments with two experimental replicates are plotted. Cultures without pCdrA exhibited a background of 145 ±3 (x1000). Statistically significant differences in fluorescence were detected between wild type and *ΔrsmIE* at 7 h (Student’s *t* test; *P*< 0.05).

**Table S1.** Primers used in this work.

| Primer name | Sequence (5´→3´)^a,b,c^ | Use |
| --- | --- | --- |
| qRTRpoSF  qRTRpoSR | GGCTCTCAGTAAAGAAGTGCCG  GTGGCATCGAGCGCCCTG | qRT_PCR (*rpoS*, PP_1623) |
| qRTCfcRF  qRTCfcRR | CTCGACGTTGTGCAACTGAC  TCGTGTACGCGGTAGATCTG | qRT_PCR (*cfcR*, PP_4959) |
| qRT16SF  qRT16SR | AAAGCCTGATCCAGCCAT  GAAATTCCACCACCCTCTACC | qRT_PCR (16S rRNA) |
| 4959KpnIF  4959rev2 | GGTACCAGCGCTACCTGAAA  GCATGCGTTATGTGACCGACTTCAA | *P_cfcR_::‘lacZ* transcriptional fusion in pMIR200 |
| 4959KpnIF  ruprev | GGTACCAGCGCTACCTGAAA  agatctgcatcCATgctgcTTCTC | *cfcR’-‘lacZ* translational fusion in pMIR219 |
| 4959KpnIF  ruprev2 | GGTACCAGCGCTACCTGAAA  agatctaaCATgctgcTTCTCTGttatgtg | *cfcR*_(bmod)_*’–‘lacZ* translational fusion in pMIR220 |
| ATTO700-labeled | AAAAAAAACCCCCCCCC | InfraRedDye-labeled DNA primer |
| PT7cfcRF  PT7cfcRR | TTTTCTGCAGTAATACGACTCACTATAGGATAGCGTTCTGATTGCATT  *AAAAAAAACCCCCCCCC*GTCGTCTACCACCAGAAGAA | DNA-CfcR(ab) template used for the synthesis of labeled RNA-CfcR(ab); hybridized with the labeled ATTO700 oligo |
| PT7cfcRF  PT7cfcRR2 | TTTTCTGCAGTAATACGACTCACTATAGGATAGCGTTCTGATTGCATT  GTCGTCTACCACCAGAAGAA | DNA-CfcR(ab) template used for the synthesis of RNA-CfcR(ab) specific unlabeled competitor (SUC) |
| PT7Loop1F  PT7Loop1R | TTTTCTGCAGTAATACGACTCACTATAGGATAGCGTTCTGATTGCATT  *AAAAAAAACCCCCCCCC*GACTTCAAAATACGGTTGGA3 | DNA-CfcR(a) template used for the synthesis of labeled RNA-CfcR(a); hybridized with labeled ATTO700 oligo |
| PT7Loop1F  PT7Loop1R2 | TTTTCTGCAGTAATACGACTCACTATAGGATAGCGTTCTGATTGCATT  GACTTCAAAATACGGTTGGA | DNA-CfcR(a) template used for the synthesis of RNA-CfcR(a) specific unlabeled competitor (SUC) |
| PT7Loop2F  PT7Loop2R | TTTTCTGCAGTAATACGACTCACTATAGGTCCAACCGTATTTTGAAGTC  *AAAAAAAACCCCCCCCC*GTCGTCTACCACCAGAAGAA | DNA-CfcR(b) template used for the synthesis of labeled RNA-CfcR(b); hybridized with labeled ATTO700 oligo |
| PT7Loop2F  PT7Loop2R2 | TTTTCTGCAGTAATACGACTCACTATAGGTCCAACCGTATTTTGAAGTC  GTCGTCTACCACCAGAAGAA | DNA-CfcR(b) template used for the synthesis of RNA-CfcR(b) specific unlabeled competitor (SUC) |

^a^ Restriction sites inserted in the primer for the cloning strategy are underlined

^b^T7 polymerase promoter is double underlined

^c^ Italics indicate the sequence used to hybridize with labeled ATTO700

**References**

Zuker, M. (2003) Mfold web server for nucleic acid folding and hybridization prediction. *Nucleic Acids Res* **31:** 3406-3415.
